# Supplementary material for: High spatial fidelity among foraging trips of Masked Boobies from Pedro Cays, Jamaica
Source: PLoS One. 2020 Apr 27;15(4):e0231654. doi: 10.1371/journal.pone.0231654 (PMC7186003; doi:10.1371/journal.pone.0231654)
Supplement: S1 Fig — Note missing decimals in plots of mean sea surface salinity and variance of sea surface salinity. (DOCX) [file pone.0231654.s001.docx]

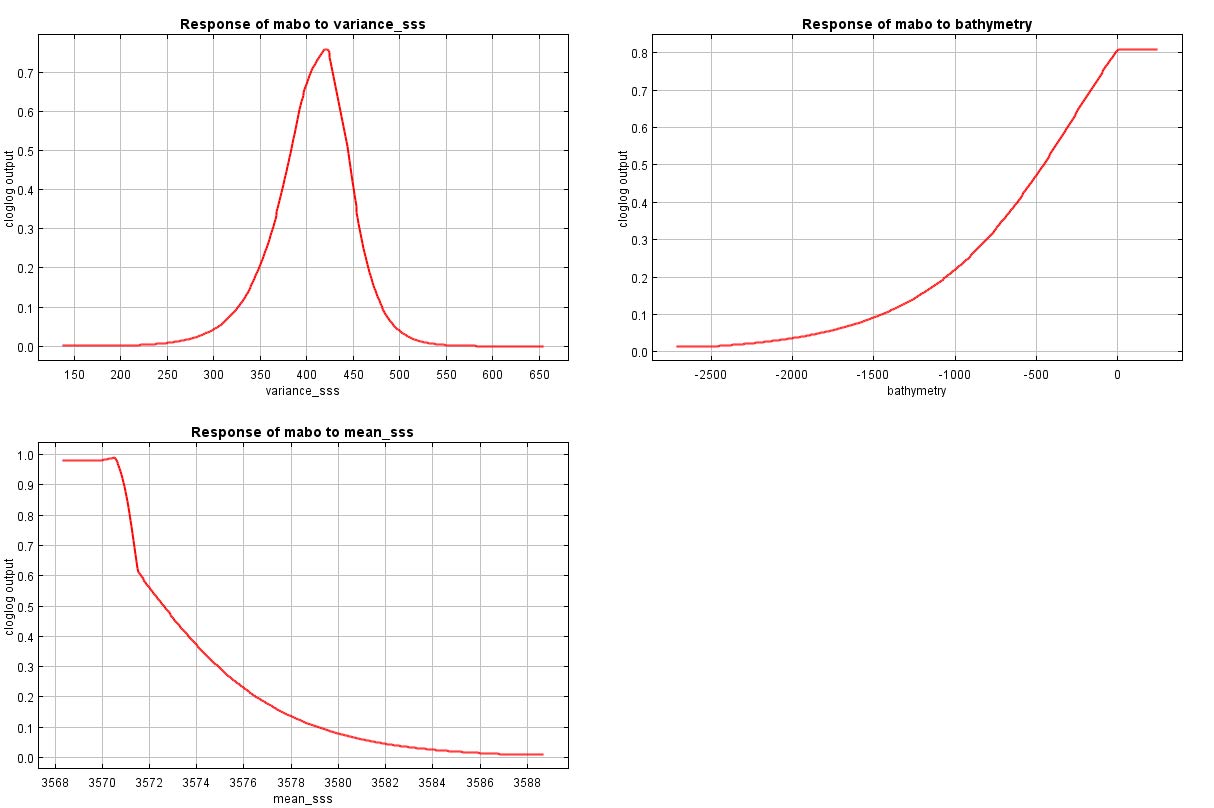


Figure S1. Estimated relationships between oceanographic variables identified via Maxent and Masked Booby habitat suitability. Note missing decimals in plots of mean sea surface salinity and variance of sea surface salinity.
